# Bioactive Properties of Carotenoids in Ocular Diseases: Antioxidant, Anti-Inflammatory, and Neuroprotective Effects

**DOI:** 10.3390/nu18152467

**Published:** 2026-07-29

**Authors:** Justyna Łapińska, Klaudia Kasperczuk, Agata Koba, Emilia Kiełczyńska, Alicja Forma, Joanna Dolar-Szczasny, Robert Rejdak, Jolanta Flieger, Grzegorz Teresiński, Jacek Baj

**Affiliations:** 1 Student Scientific Society of Forensic Medicine, Medical University of Lublin, Jaczewskiego 8b, 20-090 Lublin, Poland; lapinskaju@gmail.com (J.Ł.); klaudiakasperczukk@gmail.com (K.K.); agata.koba22@gmail.com (A.K.); 2 Faculty of Medicine, Wroclaw Medical University, Ludwik Pasteur 1, 50-367 Wrocław, Poland; kielczynskae@gmail.com; 3 Chair and Department of Forensic Medicine, Medical University of Lublin, Jaczewskiego 8b, 20-090 Lublin, Poland; grzegorz.teresinski@umlub.edu.pl; 4 Department of General and Pediatric Ophthalmology, Medical University of Lublin, 20-079 Lublin, Poland; joannadolarszczasny@umlub.edu.pl (J.D.-S.); robert.rejdak@umlub.edu.pl (R.R.); 5 Department of Analytical Chemistry, Medical University of Lublin, Chodźki 4A, 20-093 Lublin, Poland; jolanta.flieger@umlub.edu.pl; 6 Department of Correct, Clinical, and Imaging Anatomy, Medical University of Lublin, Jaczewskiego 4, 20-090 Lublin, Poland; jacek.baj@umlub.edu.pl

**Keywords:** carotenoids, ocular diseases, oxidative stress, retinal neuroprotection, ocular inflammation

## Abstract

Ocular diseases are a significant public health problem worldwide and represent one of the leading causes of disability. The prevalence of visual impairment is steadily increasing, largely driven by the ageing population. Oxidative stress, chronic inflammation and neurodegenerative processes underlie the pathogenesis of many eye diseases, including age-related macular degeneration (AMD), diabetic retinopathy, glaucoma, and cataracts, contributing to progressive vision loss and functional impairment. Carotenoids such as lutein, zeaxanthin, meso-zeaxanthin, β-carotene, lycopene, and astaxanthin exhibit multidirectional biological effects, including antioxidant, anti-inflammatory, and neuroprotective properties. These compounds are selectively accumulated in the tissues of the eye, especially in the retina and macula, where they neutralise reactive oxygen species, modulate inflammatory pathways, stabilise mitochondrial function and support the survival and function of retinal ganglion cells and photoreceptors. The aim of our review was to provide a comprehensive review of current data regarding the mechanisms of action of carotenoids and their potential clinical significance in the prevention and treatment of retinal, optic nerve, lens, and eye surface diseases, including dry eye syndrome and Meibomian gland dysfunction. We identified results of available experimental and clinical research and outlined that an adequate supply of carotenoids in the diet or in the form of supplementation may support the protection of eye structures against oxidative and inflammatory damage, improve visual performance and potentially slow disease progression. However, high-quality prospective clinical trials are necessary to conclusively assess their therapeutic efficacy and establish evidence-based recommendations for clinical practice.

## 1. Introduction

Eye health and good vision are crucial to many aspects of life, health, and social and economic development, whilst vision problems negatively impact quality of life and limit equal access to education and career opportunities [1,2,3]. Ocular diseases are a significant public health problem worldwide and represent one of the leading causes of disability. The leading causes of blindness and visual impairment are cataracts, glaucoma, diabetic retinopathy, age-related macular degeneration (AMD) and refractive errors [4,5,6,7].

Current figures show that 43.3 million people are living with blindness, and 295 million people are living with moderate or severe visual impairment. It is estimated that by 2050, the number of people living with blindness will rise to 61 million, and the number of people with moderate or severe visual impairment could rise to over 834 million. This increase is mainly due to an ageing population and an increase in the overall population [4,8,9].

Chronic inflammation, oxidative stress, and tissue damage underlie the pathogenesis of many eye diseases, including ocular disorders of the anterior segment and degenerative retinal diseases. When the balance between reactive oxygen species (ROS) and antioxidant mechanisms is disrupted, an excess of ROS causes chronic oxidative stress, apoptosis and cellular damage in the retina and the anterior segment tissue. Oxidative stress is closely linked to inflammation through the activation of pro-inflammatory pathways by ROS, as well as to mitochondrial dysfunction, deoxyribonucleic acid (DNA) damage, lipid peroxidation, protein modification and apoptosis [10,11,12,13,14].

Chronic oxidative damage and impaired antioxidant mechanisms are key factors in the development of retinal neurodegeneration in AMD. Carotenoids such as lutein, zeaxanthin, and meso-zeaxanthin, which are lipid-soluble compounds, are potent antioxidants and anti-inflammatory mediators in the retina; as such, they may exert neuroprotective effects in the retina and prevent neurodegeneration by neutralising free radicals. The mechanisms of carotenoids include attenuating ROS production, inhibiting the Tumor Necrosis Factor alpha (TNF-α) and Vascular Endothelial Growth Factor (VEGF) pathways, suppressing apoptosis, and reducing the production of inflammatory markers. These carotenoids are localised in the macula, with their spatial distribution across various layers of the retina, and their levels are strongly associated with the protection of retinal tissue and visual function. Lutein and zeaxanthin are not synthesised by the human body and must be obtained from the diet; sources include spinach, kale, green leafy vegetables, sweetcorn and egg yolks. Although clinical benefits of increasing macular pigment levels through carotenoid supplementation have been demonstrated, the comprehensive neuroprotective potential of carotenoids in the treatment of AMD has not yet been fully explored, which justifies the need for a systematic review and further research [15,16,17,18].

The aim of this article is to provide a comprehensive review of current data regarding the mechanisms of action of carotenoids and their potential clinical significance in the prevention and treatment of diseases of the retina, optic nerve, lens and eye surface, including dry eye syndrome and Meibomian gland dysfunction.

The paper analyzes available experimental and clinical research and aims to outline that an adequate supply of carotenoids in the diet or in the form of supplementation may support the protection of eye structures against oxidative and inflammatory damage, improve visual performance and potentially slow disease progression. However, high-quality prospective clinical trials are necessary to conclusively assess their therapeutic efficacy and establish evidence-based recommendations for clinical practice.

## 2. Material and Methods

This manuscript was prepared as a structured narrative review aimed at providing a comprehensive synthesis of current evidence regarding the biological properties of carotenoids and their role in ocular health and disease. A structured literature search was conducted using PubMed/MEDLINE, Scopus, and Google Scholar for English-language articles. Particular emphasis was placed on studies published within the last 10–15 years, although older landmark studies were included when considered essential for historical context or for understanding key biological mechanisms published up to January 2026. Representative PubMed search strings included: (“carotenoids” OR lutein OR zeaxanthin OR meso-zeaxanthin OR astaxanthin OR lycopene OR crocin) AND (“ocular diseases” OR retina OR retinal OR macula OR ophthalmology); (“carotenoids” OR lutein OR zeaxanthin) AND (“oxidative stress” OR inflammation OR neuroprotection OR mitochondrial dysfunction); (“lutein” OR zeaxanthin OR carotenoids) AND (“age-related macular degeneration” OR diabetic retinopathy OR retinal ischemia OR Stargardt disease); and (“carotenoids” OR lutein OR zeaxanthin) AND (glaucoma OR optic neuropathy OR cataract OR dry eye disease OR Meibomian gland dysfunction). Similar search strategies adapted to database-specific syntax were applied in Scopus and Google Scholar. Searches were updated prior to manuscript submission to include the most recent eligible publications.

Study selection was performed in several stages. Initially, titles and abstracts retrieved through the database searches were screened for relevance to the topic of carotenoids and ocular health. Publications were considered potentially eligible if they addressed the biological mechanisms of carotenoids, their ocular distribution, or their role in retinal, optic nerve, lens, or ocular surface diseases. Subsequently, full texts of potentially relevant articles were reviewed. Priority was given to original experimental studies, clinical trials, epidemiological studies, systematic reviews, meta-analyses, and landmark publications that significantly contributed to the understanding of carotenoid-mediated ocular protection. Additional relevant studies were identified through manual screening of reference lists from selected articles. When multiple publications reported overlapping findings, preference was given to the most comprehensive, recent, or methodologically robust source.

Inclusion criteria: studies were prioritized if they (i) investigated the biological mechanisms of carotenoids relevant to ocular tissues, including antioxidant, anti-inflammatory, neuroprotective, mitochondrial, or photoprotective effects; (ii) evaluated the role of carotenoids in retinal, optic nerve, lens, ocular surface, or developmental eye disorders; (iii) provided clinical, epidemiological, or experimental evidence regarding dietary carotenoid intake, supplementation, ocular distribution, or visual outcomes; and/or (iv) represented landmark clinical trials, systematic reviews, meta-analyses, or highly cited publications contributing substantially to the current understanding of carotenoid-related ocular protection.

Exclusion criteria: studies were excluded or deprioritized when they (i) focused primarily on non-ocular outcomes without direct relevance to eye health; (ii) lacked sufficient methodological detail or scientific rigor; (iii) represented duplicate or substantially overlapping datasets without providing additional insights; or (iv) consisted of conference abstracts, editorials, opinion papers, or anecdotal reports without adequate supporting evidence.

**Figure 1:**
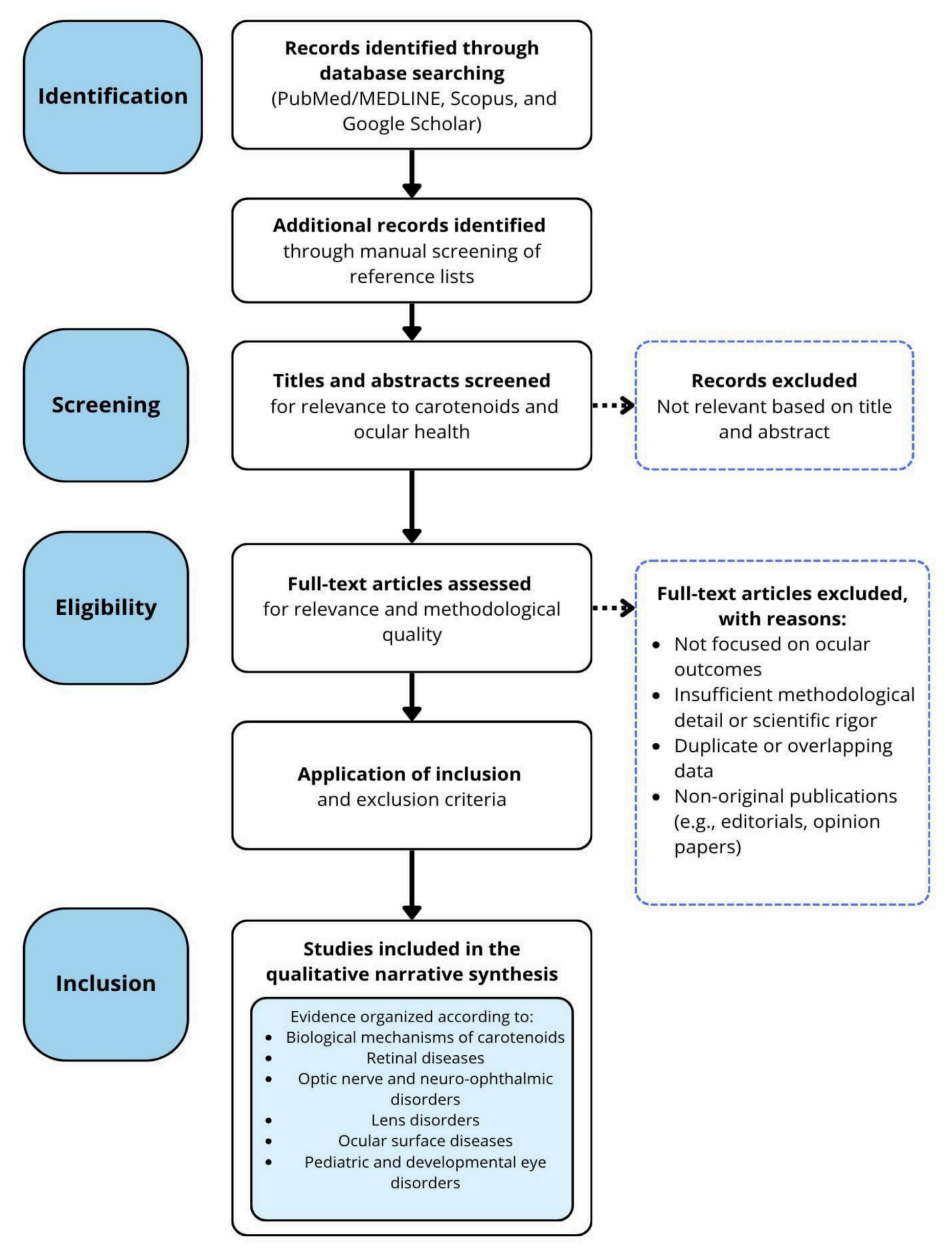
Literature search and study selection process.

For the included literature, information regarding carotenoid type, biological mechanisms, ocular targets, disease-specific findings, and reported clinical outcomes was extracted and synthesized qualitatively. Due to the heterogeneity of study designs, populations, interventions, and outcome measures, findings were summarized as a narrative qualitative synthesis organized according to major mechanistic pathways and ophthalmological disease categories. The manuscript represents a qualitative narrative review; therefore, exact study counts at each screening stage were not qualitatively tracked. The literature selection process, search parameters and inclusion/exclusion criteria are summarized in a flow diagram (Figure 1).

## 3. Overview of Carotenoids Relevant to Ocular Health

Carotenoids are a diverse group of lipophilic natural pigments synthesized primarily by plants, algae, and certain microorganisms. Structurally, they belong to the isoprenoid class and perform important biological functions. Due to the presence of oxygen atoms in the molecule, they are divided into two main groups: xanthophylls and carotenes [19,20].

Xanthophylls are a subgroup of carotenoids that contain oxygen atoms in the form of hydroxyl or epoxide groups, which makes them more polar than carotenes. This characteristic influences their localization in biological membranes and their bioavailability. The most important xanthophylls include lutein, zeaxanthin, and meso-zeaxanthin. Lutein and zeaxanthin are structural isomers, while meso-zeaxanthin is produced primarily in the human body through the conversion of lutein and accumulates in the macula of the retina [21,22,23]. The main dietary sources of xanthophylls are green leafy vegetables, such as spinach and kale, where these compounds are found in chloroplasts. Egg yolk is also a significant source of lutein and zeaxanthin, characterized by high bioavailability of these carotenoids due to the presence of lipids that facilitate their absorption [22,24].

Carotenes constitute the second major group of carotenoids and are hydrocarbon compounds devoid of oxygen atoms, which gives them a strongly hydrophobic nature. Their structure is based exclusively on isoprene chains. The most important members of this group include β-carotene and lycopene. β-carotene acts as a provitamin A and can be converted in the body to retinol, whereas lycopene does not exhibit this activity but is characterized by high biological reactivity resulting from the presence of numerous conjugated double bonds [25,26]. β-carotene is found primarily in orange and yellow vegetables and fruits, such as carrots, pumpkin, and sweet potatoes. Lycopene, on the other hand, is the predominant carotenoid in tomatoes and tomato products, and its bioavailability increases after heat treatment and in the presence of dietary fat [22,25]. In addition to plant-derived carotenoids, there are also marine-derived carotenoids, including astaxanthin. This compound contains both hydroxyl and ketone groups, which distinguishes it from other carotenoids and influences its biological properties. Astaxanthin is synthesized by microalgae and then accumulates in marine organisms such as salmon, shrimp, and crabs [27,28].

### Absorption, Transport, and Ocular Distribution

As lipophilic compounds, carotenoids require the presence of dietary lipids for effective absorption in the gastrointestinal tract. This process occurs primarily in the small intestine and involves the release of carotenoids from the food matrix, their incorporation into mixed micelles, and transport across enterocytes. Carotenoid absorption occurs partly via passive diffusion; however, specific membrane transporters, such as scavenger receptor class B type I (SR-BI) and Niemann-Pick C1-Like 1 (NPC1L1), also play a significant role [29,30]. After absorption, carotenoids are incorporated into chylomicrons and transported via the lymphatic system into the systemic circulation. They are then redistributed among various lipoprotein fractions, primarily Low-Density Lipoprotein (LDL) and High-Density Lipoprotein (HDL), which enables their delivery to peripheral tissues (Reboul, 2019 [29]). It is worth noting that xanthophylls, due to their higher polarity, exhibit a greater affinity for the HDL fraction, whereas carotenes are more frequently transported in LDL [29,31].

A distinctive feature of certain carotenoids, particularly lutein, zeaxanthin, and meso-zeaxanthin, is their selective accumulation in the retina, specifically in the macula. This process results from the presence of specific binding proteins, such as StAR-related lipid transfer domain protein 3 (StARD3) for lutein and glutathione S-transferase Pi 1 (GSTP1) for zeaxanthin, which are responsible for the uptake and retention of these compounds in eye tissues [21,24]. The selective distribution of carotenoids within the eye has significant functional importance, as these compounds are concentrated in the macula, where they act as blue light filters and help protect retinal structures from oxidative stress. The high concentration of lutein and zeaxanthin in this structure reflects their specific chemical properties as well as their transport and uptake mechanisms [21,23,24].

It should be noted that carotenoid bioavailability exhibits considerable inter-individual variability that cannot be explained solely by dietary intake or lipid consumption. Host-related factors, including age, gastrointestinal function, metabolic status, and gut microbiota composition, significantly influence carotenoid absorption and tissue distribution. Recent evidence suggests that intestinal microorganisms may affect carotenoid release from the food matrix, biotransformation, and micellar incorporation, thereby modulating systemic availability. Genetic factors also contribute substantially to variability in carotenoid metabolism. Polymorphisms in genes involved in intestinal uptake and metabolism, particularly SCARB1 and BCO1, have been associated with differences in circulating carotenoid concentrations and conversion efficiency of provitamin A carotenoids. Variants in other transport-related genes, including CD36 and ABCG5/ABCG8, may further influence absorption efficiency and tissue accumulation. These findings indicate that individuals may respond differently to similar dietary carotenoid intakes or supplementation regimens. Such observations have contributed to the development of the precision nutrition concept, which aims to tailor dietary recommendations according to an individual’s genetic background, metabolic characteristics, and microbiome profile. In the future, precision nutrition approaches may improve the effectiveness of carotenoid-based dietary interventions and help optimize strategies for the prevention and management of ocular diseases [29,30].

## 4. Mechanisms of Action of Carotenoids in the Eye

**Figure 2:**
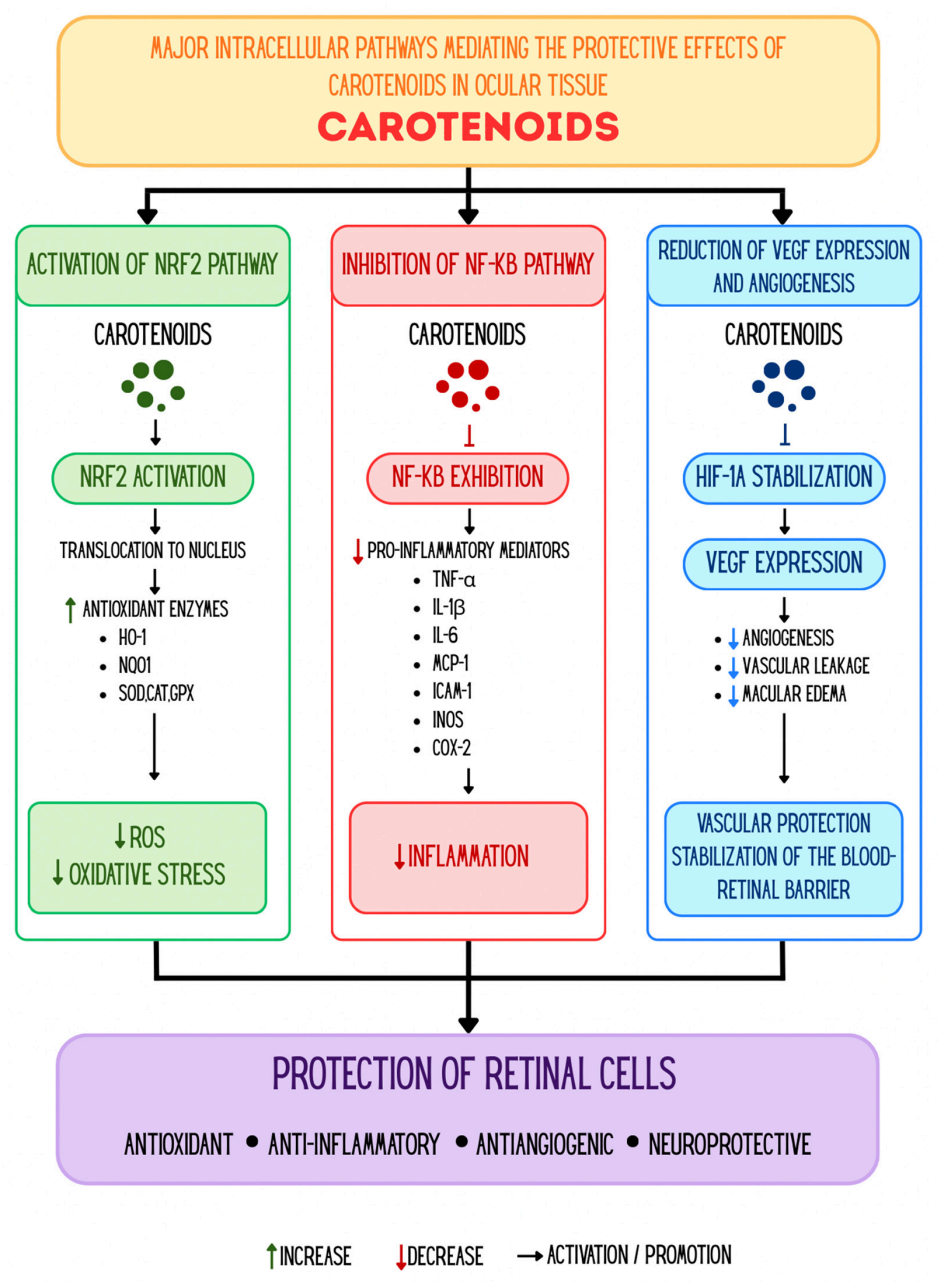
Major intracellular pathways mediating the protective effects of carotenoids in ocular tissue. Nrf2—Nuclear Factor Erythroid 2-Related Factor 2, HO-1—Heme Oxygenase-1, NQO-1—NAD(P)H Quinone Oxidoreductase 1, SOD—Superoxide Dismutase, ROS—Reactive Oxygen Species, NF-κB—Nuclear Factor Kappa B, TNF-α—Tumor Necrosis Factor Alpha, IL-1β—Interleukin-1 Beta, IL-6—Interleukin-6, MCP-1—Monocyte Chemoattractant Protein-1, ICAM-1—Intercellular Adhesion Molecule-1, iNOS—Inducible Nitric Oxide Synthase, COX-2—Cyclooxygenase-2, VEGF—Vascular Endothelial Growth Factor, HIF-1α—Hypoxia-Inducible Factor-1 Alpha.

Oxidative stress, mitochondrial dysfunction, apoptosis, and inflammatory processes represent common pathogenic mechanisms underlying many ocular diseases. Preclinical evidence indicates that Macular carotenoids exhibit multifaceted protective effects in the retina, including antioxidant, anti-inflammatory, and neuroprotective mechanisms [10,17]. The mechanisms of action of carotenoids in the eye are illustrated in Figure 2.

### 4.1. Antioxidant Effects

Free radicals are unstable, highly reactive molecules containing unpaired electrons that readily interact with cellular components. This group primarily includes ROS, which are generated both endogenously—mainly in mitochondria during metabolic processes—and as a result of environmental factors. Endogenous ROS are predominantly formed in mitochondria due to electron leakage in the respiratory chain, leading to oxygen reduction and the formation of superoxide anion. Additionally, ROS are generated by oxidative enzymes such as reduced form of Nicotinamide Adenine Dinucleotide Phosphate (NADPH) oxidases, xanthine oxidase, and lipoxygenases, particularly in endothelial cells and phagocytes [10]. Current evidence indicates that the role of mitochondria in oxidative stress extends beyond the classical mechanism of electron leakage in the respiratory chain. Dysregulation of mitochondrial dynamics, including disturbances in fusion and fission processes as well as impaired mitophagy, leads to the accumulation of damaged mitochondria and increased ROS production. Moreover, cytoplasmic sources of ROS, including NADPH oxidase, induce secondary mitochondrial dysfunction, creating a positive feedback loop. Epigenetic changes and disturbances in redox homeostasis further exacerbate this process. Consequently, mitochondria act both as a source and a target of oxidative stress [32]. Under physiological conditions, ROS levels are tightly regulated and play an essential role in cellular signaling. However, excessive ROS production or insufficient antioxidant defenses disrupt redox balance. As a result, ROS initiate a cascade of oxidative reactions involving damage to lipids, proteins, and nucleic acids, ultimately leading to cellular dysfunction and activation of apoptotic pathways [10]. The retina is particularly susceptible to oxidative stress due to the interplay of multiple factors promoting ROS generation. Key contributors include its high metabolic activity and oxygen consumption, the abundance of mitochondria as the primary ROS source, and the high content of polyunsaturated fatty acids prone to peroxidation. Additionally, constant exposure to light, especially short-wavelength light, enhances photo-oxidative processes. Consequently, the retina is especially vulnerable to redox imbalance and associated cellular damage [10,17,33,34]. Given the high susceptibility of the retina to oxidative stress, protective mechanisms—including the presence of macular carotenoids—are of particular importance [17,24]. Macular carotenoids, such as lutein, zeaxanthin, and meso-zeaxanthin, possess the ability to selectively absorb blue light due to their chemical structure, thereby acting as a natural optical filter and limiting the initiation of photo-oxidative processes in the retina [24,33,34]. Among known carotenoids, lutein exhibits a particularly high capacity for blue light absorption, highlighting its key role in retinal protection [34]. Filtering short-wavelength radiation reduces the formation of light-induced ROS [17,24,33]. Importantly, the protective effects of carotenoids are not limited to blue light filtration. Experimental studies have demonstrated that even at low concentrations—insufficient to produce an optical filtering effect—lutein protects the retina through direct scavenging of free radicals. These findings suggest that macular carotenoids act both at the preventive stage, by absorbing radiation that initiates oxidative stress, and at the secondary stage, by neutralizing already formed free radicals [34]. Experimental studies have shown that, At the cellular level, these compounds act as antioxidants by neutralizing free radicals and inhibiting lipid peroxidation in photoreceptor membranes [17,24,33]. Macular carotenoids are oriented across the lipid bilayer of cell membranes, enabling effective protection of polyunsaturated fatty acids from peroxidation [17,24,34]. An important photoprotective mechanism is also the reduction in A2E photooxidation—a component of lipofuscin accumulating in retinal pigment epithelium (RPE) cells—which exhibits strong cytotoxic effects upon light activation. This is particularly relevant in the context of retinal degenerative processes [34]. However, it should be noted that the antioxidant activity of carotenoids is associated with their oxidation, and the resulting products may exhibit both protective and potentially cytotoxic effects, indicating a complex and condition-dependent biological activity. While the antioxidant activity of lutein and zeaxanthin is mainly due to their ability to scavenge ROS and quench singlet oxygen, their biological activity seems to be strongly dependent on the local oxidative environment. When oxidative stress is increased, such as in cases of prolonged light exposure, high oxygen tension, and excessive photooxidative stress in retinal tissues, carotenoids can be oxidatively degraded to aldehydes, endoperoxides, and other oxidation products. Data from experimental studies suggest that photooxidation-derived metabolites of lutein may exhibit not only enhanced antioxidant activity but also greater cytotoxic potential than their parent compound. Human clinical studies have shown that supplementation with lutein and zeaxanthin at doses used in clinical practice, including AREDS2 formulation (10 mg lutein and 2 mg zeaxanthin daily), is generally considered safe and well tolerated. Up to now, a clear Lowest Observed Adverse Effect Level (LOAEL) or No Observed Adverse Effect Level (NOAEL) has not been established for lutein, as clinical studies have not revealed significant adverse effects associated with high intake. Moreover, the European Food Safety Authority (EFSA) considers synthetic zeaxanthin to be safe at doses up to 0.75 mg/kg body weight/day. Nevertheless, given the context-dependent nature of carotenoid oxidation and redox homeostasis, caution may be justified in the context of prolonged high-dose supplementation. The therapeutic effects of carotenoids should be understood in a balanced dose-dependent context of antioxidant protection and possible biological risks of context-dependence. Human clinical studies have shown that macular pigments also influence visual quality by reducing optical phenomena such as glare, light scatter, and chromatic aberration. By filtering short-wavelength light, they improve contrast sensitivity and visual discrimination, resulting in enhanced image perception. Increased concentrations of these carotenoids in the macula are associated with improved visual performance and reduced susceptibility to glare. Macular carotenoids function as part of an integrated antioxidant system, in which their effectiveness depends on interactions with other antioxidants, indicating synergistic rather than isolated activity [24]. Their biological efficacy is further influenced by specific binding proteins that enable their localization in regions of high oxidative stress [17,24]. Collectively, the available evidence suggests that macular carotenoids constitute a multi-level protective system for the retina, acting both in the prevention and neutralization of oxidative damage [17,24,35]. Studies in cellular and animal models have shown that Dysfunction of mitophagy and disturbances in mitochondrial homeostasis in RPE cells play a significant role in the pathogenesis of AMD, inducing oxidative stress, activation of inflammatory pathways, and epithelial–mesenchymal transition (EMT). Studies in experimental models suggest that, carotenoids, due to their antioxidant properties, may exert protective effects by modulating these pathophysiological processes [36]. Another mechanism through which carotenoids act in the eye involves anti-inflammatory pathways. Evidence indicates that oxidative stress and inflammation form a mutually reinforcing cycle leading to retinal damage, whereas lutein disrupts this cycle by simultaneously reducing both processes, thereby exerting a protective effect [10,35].

### 4.2. Anti-Inflammatory Pathways

It should be emphasized that most of the evidence regarding the molecular mechanisms underlying the antioxidant activity of carotenoids is based on in vitro studies and animal models. In contrast, clinical studies in humans mainly demonstrate the beneficial effects of supplementation on the functional and structural parameters of the eye, while direct confirmation of the activation of the described molecular pathways remains limited [22,37].

RPE cells constitute a major source of inflammatory mediators within the retina, including cytokines, such as interleukin 6 (IL-6), interleukin 1β (IL-1β), chemokines such as interleukin 8 (IL-8), Monocyte Chemoattractant Protein 1 (MCP-1), adhesion molecules, including Intercellular Adhesion Molecule-1 (ICAM-1), and pro-inflammatory enzymes, such as inducible Nitric Oxide Synthase (iNOS). IL-1β plays a key role in initiating the inflammatory response by activating RPE cells and enhancing the production of inflammatory mediators. This process is primarily regulated by Nuclear Factor kappa B (NF-*κ*B) and Mitogen-Activated Protein Kinases (MAPK) such as Extracellular Signal-Regulated Kinases (ERK) and c-Jun N-terminal Kinases (JNK), signaling pathways responsible for the expression of inflammatory genes. Preclinical studies using cellular and animal models have demonstrated that lutein and other xanthophylls inhibit the activation of these pathways by suppressing NF-*κ*B phosphorylation and MAPK, leading to reduced levels of IL-6, IL-8, MCP-1, ICAM-1, and iNOS. These preclinical findings indicate that lutein exerts anti-inflammatory effects both through direct modulation of signaling pathways and indirectly by reducing oxidative stress. However, most evidence suggesting modification of the NF-*κ*B and MAPK pathways derives from in vitro research and experimental animal models, whereas direct validation of these molecular effects in humans remains restricted [33,37]. Preclinical studies in animals have indicated that lutein lowers retinal VEGF expression, which suggests a potential involvement in controlling vascular permeability and pathological angiogenesis. In a diabetic mouse model, lutein also protected the integrity of the blood–retinal barrier by upregulating occludin expression, maintaining tight junctions and decreasing retinal vascular leakage. These findings from animal studies suggest that lutein has a protective effect on vascular integrity, because disruption of the junctions contributes to increased vascular permeability during retinal inflammation. However, direct evidence of these pathways in human retinal tissue is lacking [38]. In vitro studies have shown that, lutein inhibits microglial activation, limiting their transition from a resting to a pro-inflammatory phenotype. A reduction in reactive microglial cells indicates a significant anti-inflammatory effect of lutein in the retina [34,38]. Human clinical studies suggest that lutein supplementation may modify systemic inflammation by decreasing circulating complement factor D, a major enzyme of the alternative complement pathway. However, this observation is limited by clinical evidence and the molecular and therapeutic importance needs additional validation [33]. Moreover, the observed decrease in carotenoid levels during ongoing inflammation may reflect their consumption in antioxidant and immunoregulatory processes. Epidemiological studies have demonstrated an inverse relationship between serum lutein levels and inflammatory markers such as soluble ICAM-1 and C-reactive protein (CRP). Although these findings support a potential role of lutein in modulating systemic inflammatory responses, they do not establish a causal relationship or directly confirm regulation of NF-κB signaling in humans [34].

### 4.3. Neuroprotective and Mitochondrial Effects

Beyond their antioxidant and anti-inflammatory properties, available evidence suggests that macular carotenoids exhibit a range of additional biological functions essential for maintaining the structural and functional integrity of the retina and the visual system. Studies in cellular and animal models suggest that they may participate in intercellular communication by influencing gap junction function, which plays a key role in maintaining cellular homeostasis [33]. Preclinical studies have shown that Lutein exerts neuroprotective effects by reducing oxidative stress, inhibiting ERK pathway activation, and protecting synaptic proteins such as synaptophysin, thereby preserving proper neuronal communication in the retina. Additionally, animal studies have shown that, lutein prevents the decline of brain-derived neurotrophic factor (BDNF), supporting neuronal survival. These effects contribute to the preservation of visual function, as confirmed by electroretinographic studies in animal models. Animal studies have shown that lutein protects retinal ganglion cells and inner retinal layers from degeneration induced by pathological processes. This is particularly important, as retinal neurons lack regenerative capacity, and their loss is irreversible [35]. Macular carotenoids also exhibit a characteristic distribution within retinal structures—beyond the fovea, high concentrations are observed in the inner plexiform layer, suggesting their role in visual signal transmission. Importantly, lutein is the predominant carotenoid in the visual cortex of the brain, and its concentration strongly correlates with retinal levels. This suggests a functional relationship between carotenoid status in the eye and the central nervous system. Consequently, the presence of lutein and zeaxanthin in both the retina and the brain indicates their potential role in protecting neuronal structures rich in polyunsaturated fatty acids and in optimizing visual signal transmission [33]. Experimental studies indicate that the antioxidant properties of macular carotenoids may contribute to functional benefits. Human clinical studies have demonstrated improvements in visual function following supplementation.

Human clinical studies have shown that supplementation with lutein and zeaxanthin increases macular pigment optical density (MPOD), which is associated with improvements in visual function parameters such as visual acuity and contrast sensitivity. The magnitude of these effects remains moderate and depends on dose, duration of supplementation, and individual variability. While laboratory investigations have discovered various molecular processes underpinning the neuroprotective effects of carotenoids, clinical trials have mostly shown improvement in MPOD, visual acuity, and contrast sensitivity, instead of directly confirming these intracellular pathways. These results support the hypothesis that carotenoids play a significant role in reducing photooxidative damage and maintaining normal retinal function; however, the molecular mechanisms responsible for the observed clinical benefits have so far been confirmed mainly in preclinical studies [33,34]. The key properties of major carotenoids, including their molecular targets, mechanisms of action, and associated ocular diseases, are summarized in Table 1.

### 4.4. Translational Challenges and Future Perspectives

However, despite increasing evidence for the beneficial benefits of carotenoids on ocular health, significant translational obstacles remain. Most of the postulated molecular processes, such as regulation of Nrf2, NF-κB, MAPK and mitochondrial signaling pathways, have been demonstrated mainly in in vitro studies and experimental animal models thus far [35,37].

In contrast, clinical trials have focused mostly on functional outcomes such as macular pigment optical density (MPOD) alterations, visual acuity and contrast sensitivity rather than demonstrating these intracellular processes in human retinal tissue. Therefore, mechanistic discoveries from experimental models should be extrapolated to clinical practice with caution [33,39].

One of the significant limitations is the high heterogeneity of clinical research. The variable research populations, baseline nutritional status, disease stage, formulation of carotenoids, dose, duration of supplementation, and outcome measures make it difficult to compare the results directly and may explain some of the variation in clinical responses. Additionally, interindividual differences in carotenoid absorption, transport and metabolism, which may be affected by genetic factors such as polymorphisms in carotenoid transport proteins, may influence treatment efficacy and should be considered in future studies [22].

Future studies should be well-designed large-scale randomized controlled trials with molecular biomarkers and clinical endpoints to better understand the connection between carotenoid-induced molecular changes and visual outcomes [22,39].

Moreover, future research should explore tailored supplementation regimens according to individual genetic and metabolic factors and assess their long-term clinical effectiveness. Addressing these obstacles will be crucial to the translation of promising experimental findings into evidence-based practice in ophthalmology [22,39].

## 5. Diseases and Disorders

### 5.1. Retinal Diseases

#### 5.1.1. Age-Related Macular Degeneration

AMD is a progressive neurodegenerative disorder affecting the macula, the central and most functional part of the retina. As the major cause of vision loss in developed countries. AMD impacts both sexes, with its prevalence increasing significantly after the age of 60. The condition is characterized by the deterioration of central visual acuity. The etiology of AMD involves aging, lipid metabolism, and oxidative stress. Genetic predisposition remains a major determinant, accounting for an estimated 46–71% of the disease risk [40,41,42]. AMD is clinically categorized into two primary forms, Dry AMD and Wet AMD. Dry AMD, accounting for most cases, is characterized by degradation of the retinal pigment epithelium and photoreceptors. This stage is defined by the buildup of drusen—sub-retinal, extracellular metabolic deposits, that disrupt nutrient transport and homeostasis of the retina. Geographic atrophy (GA) is an advanced stage of dry AMD characterized by sharply demarcated lesions across the macula, resulting from the progressive and irreversible degradation of photoreceptors, the RPE and the choriocapillaris. Currently, no treatments exist for the dry form. The second form of AMD, known as wet AMD or neovascular AMD is less common, but it can lead to rapid vision loss, due to choroidal neovascularization. Fragile new blood vessels proliferate beneath the macula, leaking blood or fluid, which results in macular edema and retinal scarring [1,2,3]. Anti-VEGF therapy remains the gold standard for treating the wet form [40,41]. While lutein and zeaxanthin primarily function as high-energy blue light filters and direct scavengers of ROS, their synergy with other nutrients improves their protective function. For instance, dietary nitrates improve the bioavailability of nitric oxide, which results in reduced lipid peroxidation in the photoreceptor outer segments and prevents the oxidative degradation of the RPE. By mitigating the oxidative load, the combination of xantophylls and nitrates inhibits the formation of drusen. These nutrients also modulate the genetic risk, the protective effects of a carotenoid and nitrate-rich diet are most pronounced in individuals with specific risk alleles (CFH and ARMS2 loci), potentially compensating for complement dysregulation and mitochondrial dysfunction in the retina. Furthermore, these nutrients improve choriocapillaris perfusion. By promoting vasodilation and enhancing blood flow to the metabolically active retinal layers, dietary nitrates work synergistically with the anti-inflammatory effects of carotenoids, which suppress pro-inflammatory cytokines such as TNF-a and IL-6 to create a stabilized environment. This is crucial for preventing the transition from dry to wet-AMD, where VEGF expression is triggered by chronic inflammation [43]. Retinal carotenoids have been shown to attenuate the formation of A2E (a pyridinium bisretinoid), a toxic product of metabolism, which contributes to the development of drusen. Through the mitigation of A2E-mediated oxidative damage, lutein and zeaxanthin and meso-zeaxanthin enhance RPE cell survival, slowing the progression of GA [44]. Large-scale clinical evidence from the Age-Related Eye Disease Studies (AREDS and AREDS2) has demonstrated the impact of nutrition on AMD progression. Data from a combined cohort of over 7700 participants reveal that a high dietary intake of lutein, zeaxanthin, and beta-carotene significantly reduced the risk of developing late-stage manifestations, such as GA and wet AMD. These carotenoids, particularly when consumed in the highest quintiles, emerged as significant protective factors for long-term retinal preservation, protection against large drusen formation, and neovascular complications. Among 33 examined nutrients, xanthophyll carotenoids remain the most statistically significant for long-term retinal preservation [43,45]. In patients with non-central GA, supplementation with 10 mg lutein and 2 mg zeaxanthin significantly decelerates the expansion of atrophic lesions toward the foveal center. The progression rate was slowed to 80.1 μm/year, compared to 114.4 μm/year in the control group, suggesting that xanthophyll carotenoids act as a “buffer”, preserving the central visual acuity even after the onset of the GA. Furthermore, the AREDS2 formulation confirmed that replacing beta-carotene with lutein and zeaxanthin maintains this protective efficacy while eliminating the increased risk of lung cancer previously observed in smokers [46]. Moreover, higher adherence to diets where lutein and zeaxanthin are key components was associated with a hazard ratio of 0.77 for late AMD, indicating a 23% reduction in overall risk [43]. Similar observations were made in epidemiological studies, which revealed that patients with AMD have a significantly lower dietary Total Antioxidant Capacity compared to healthy individuals (12.3 vs. 14.9 mmol/d), possibly due to a reduced intake of carotenoid-rich fruits and vegetables [46]. Furthermore, the significant negative correlation between the Composite Dietary Antioxidant Index and AMD prevalence reinforces the role of these pigments as a primary chemical shield against chronic oxidative damage to the retina [47,48]. Nevertheless, these observational associations must be interpreted cautiously. Such studies often suffer from limitations including small sample sizes, short follow-up durations, and a lack of standardized protocols for dietary assessment, which introduces significant heterogeneity of study populations. These findings highlight a complex interaction between diet and genetics, suggesting that individuals with specific risk variants may derive varying levels of neuroprotection from carotenoid-rich dietary patterns [43]. The effectiveness of these xanthophylls depends on how well they are absorbed. They are fat-soluble, so they need dietary fats for proper uptake and transport to the eye. The food source also matters—lutein from egg yolks, for example, is absorbed better than from supplements. Consuming healthy fats, such as omega-3, may further support macular pigment stability and help protect against oxidative stress [43,45,49]. Despite these established pathways, forming universal guidelines remains difficult due to a limited number of randomized controlled trials outside the major AREDS cohorts. The broader literature is heavily fragmented by differences in supplementation doses and variability in outcome measures.

#### 5.1.2. Diabetic Retinopathy

Diabetes mellitus (DM) is characterized by chronically elevated blood glucose levels, which drive the accumulation of advanced glycation end products (AGEs). This process is a major contributor to an increased risk of microvascular and macrovascular complications, with Diabetic Retinopathy (DR) being one of the most debilitating ocular manifestations. In the retina, xanthophylls counteract the microvascular damage caused by hyperglycemia-induced oxidative stress. Experimental models demonstrate that carotenoids, specifically zeaxanthin and lutein, mitigate retinal abnormalities by reducing mitochondrial stress and regulating genes (such as SCARB1 and BCO2), which are involved in antioxidant defense. However, relying on these experimental models highlights a significant limitation, as there is a predominance of preclinical evidence in this area. Translating these molecular mechanisms to human pathology often results in inconsistent findings across clinical studies. Sustained antioxidant supplementation involving lutein may effectively prevent the progression of DR over long-term periods. Large-scale epidemiological studies provide strong evidence for the protective role of carotenoids in DR. Data from the EPIC-InterAct study demonstrated that higher plasma concentrations of total carotenoids, specifically a-carotene, B-carotene, and lutein, are associated with a reduced risk of developing type 2 DM. Each standard deviation increase in total carotenoid levels was linked to an approximately 25% lower risk of developing diabetes [48,50]. A cross-sectional study of the Chinese urban population suggests that individual carotenoids may exert distinct protective effects. β-carotene may be a protective factor for DM, as reflected by its negative correlation with fasting glucose levels, indicating a potential role in glycemic control. In contrast, α-carotene appears to be particularly protective against diabetic retinopathy, especially among smokers [10]. Additionally, a higher intake of carotenoids and zinc has been associated with a lower prevalence of DR, pointing to a possible synergistic effect in mitigating microvascular damage induced by chronic hyperglycemia [51]. Despite these promising associations, observational and cross-sectional data must be interpreted with caution. These studies frequently face limitations such as the heterogeneity of study populations and a lack of standardized protocols for measuring dietary intake, which complicates direct comparisons. Recent advances in clinical diagnostics allow for the rapid, non-invasive assessment of a patient’s antioxidant status. Skin-based sensors can be used to estimate both AGEs and carotenoid levels. This is relevant because higher vegetable intake—reflected by an increased “Veggie score”—has been shown to correlate inversely with AGE accumulation, reinforcing the role of diet in modulating oxidative stress [52]. From a clinical perspective, the effectiveness of carotenoid supplementation appears to be highly sensitive to dosage. Increasing the dosage of lutein from 10 mg to 20 mg/day significantly compresses the timeframe for reaching target MPOD levels, reducing the time needed to reach the target from one year to less than six months [53]. While these dose-dependent effects are encouraging, establishing definitive clinical guidelines is hindered by a limited number of randomized controlled trials. The available interventional studies often suffer from small sample sizes, short follow-up durations, differences in supplementation doses, and significant variability in outcome measures. Evidence from a large-scale, nationally representative study in the United States is consistent with these findings. It demonstrated the significant protective role of systemic carotenoid status against DR. The findings indicated that higher serum concentrations of b-carotene and b-cryptoxanthin are negatively correlated with the risk of developing DR. Moreover, higher total carotenoid levels are linked to a reduced risk of multiple ocular diseases, including AMD, cataract, glaucoma. The protective effect is especially pronounced in women [54]. Overall, the data indicates the importance of systemic carotenoid levels as a modifiable determinant of reduced diabetic risk and sustained visual health.

#### 5.1.3. Retinal Ischemia and Ischemia–Reperfusion Injury

Retinal Ischemia is a pathological process involving a sudden disruption of blood flow to the retina. Ischemia–Reperfusion injury, in turn, refers to an initial interruption of blood supply followed by its subsequent restoration, which triggers a cascade of oxidative stress and neuroinflammation. Recent molecular investigations have identified specific carotenoids as agents capable of mitigating these destructive pathways.

Crocin, a bioactive water-soluble carotenoid derived from saffron, has been shown to exert a significant protective effect on Retinal Ganglion Cells. Its mechanism of action primarily involves the activation of the Sirt6–Nrf2/HO-1 signaling axis. This pathway serves as a critical defense line against both oxidative stress and endoplasmic reticulum stress within the retina. The data demonstrated that crocin suppresses apoptosis by reducing the expression of pro-apoptotic markers such as cleaved caspase-3 and Bax, inhibits neuroinflammation by lowering the levels of pro-inflammatory cytokines like TNF-a and IL-6, and preserves anatomy by maintaining the integrity of the Ganglion Cell Layer, suggesting that saffron-derived carotenoids could serve as a viable therapeutic strategy for managing ischemic retinal disorders [55].

Lycopene also plays a vital role in preserving retinal function during Ischemia–Reperfusion injury, specifically by maintaining the integrity of the Blood–Retinal Barrier (BRB). Recent in vivo and in vitro models indicate that lycopene stabilizes both the inner BRB (microvascular endothelial cells) and the outer BRB (retinal pigment epithelium). This stabilization is achieved through the modulation of the Kelch-like ECH-associated protein A (KEAP1)/Nuclear factor erythroid 2-related factor 2 (NRF2)/Antioxidant Response Element (ARE) pathway. Lycopene promotes the nuclear translocation of phosphorylated NRF2 (p-NRF2), which triggers the upregulation of phase II antioxidant enzymes, such as NADPH Quinone Oxidoreductase 1 (NQO-1) and Heme Oxygenase-1 (HO-1). The impact of lycopene has been confirmed via electroretinography, where intervention preserved scotopic b-wave amplitudes. It indicates a recovery of retinal circuitry and reduction in apoptosis across all primary retinal layers [56]. The multifaceted neuroprotective profile of crocin and lycopene highlights their potential as powerful therapeutic agents for managing retinal ischemic conditions. These carotenoids provide protection by preserving the structural integrity of the blood–retinal barrier and ensuring the functional survival of retinal ganglion cells. These compounds neutralize the oxidative burst and inflammatory cascade following reperfusion. Integrating these bioactive pigments into clinical strategies could offer a promising, non-invasive approach to mitigating the irreversible visual loss associated with ischemia–reperfusion injury.

#### 5.1.4. Retinitis Pigmentosa and Inherited Retinal Degenerations

Retinitis pigmentosa (RP) is a genetically heterogeneous group of progressive retinopathies characterized by loss of photoreceptors and subsequent atrophy of the RPE, ultimately leading to bilateral blindness. The pathogenesis of RP is complex, involving various regulatory cell death pathways, dysregulated apoptosis, together with autophagic and necrotic signaling, is responsible for the irreversible loss of photoreceptors. Clinically, RP typically manifests first as nyctalopia (loss of night vision). As the disease progresses, the visual field is lost in a concentric pattern, often referred to as “tunnel vision”. This results from the extensive loss of peripheral photoreceptors and the subsequent degeneration of central foveal cone cells, which are crucial for high-acuity vision [57].

Autosomal recessive Stargardt disease type 1 (STGD1) is an inherited blinding disorder caused by mutations in the ABCA4 gene. The ABCA4 protein functions as a transmembrane flippase in photoreceptor cells, responsible for eliminating all-trans-retinal, a toxic byproduct of the visual cycle. In the context of STGD1, the neuroprotective role of macular carotenoids extends beyond antioxidant activity. The accumulation of toxic lipofuscin fluorophores, such as A2E manifests as characteristic autofluorescent flecks in the retina. Research indicates that the presence of lutein, zeaxanthin, and meso-zeaxanthin may impair the biochemical pathways leading to A2E synthesis. By limiting the accumulation of these bisretinoids and mitigating the oxidative stress, carotenoids provide a specialized form of neuroprotection that may preserve photoreceptor integrity in patients with retinal dystrophies [43]. Clinical data suggest that high dietary intake of standard Vitamin A retinol may paradoxically correlate with poorer visual acuity in patients with ABCA4 mutations. This is because standard Vitamin A is a substrate for the formation of toxic bisretinoids, when the ABCA4 flippase is dysfunctional. Experimental models have demonstrated that this modified Vitamin A significantly slows the formation of toxic byproducts and preserves retinal function. These findings underscore the need for precision in nutritional interventions, while macular carotenoids act as a safe “chemical shield,” standard Vitamin A supplementation may require strict limitation in the STGD1 population [58].

### 5.2. Optic Nerve and Neuro Ophthalmic Disorders

#### 5.2.1. Glaucoma

Glaucoma is a chronic, irreversible group of disorders characterized by progressive optic neuropathy and degeneration of retinal ganglion cells and their axons. This damage leads to a significant reduction in visual sensitivity, typically starting in the visual field. While intraocular pressure (IOP) remains the primary modifiable risk factor, increasing evidence suggests that oxidative stress and metabolic dysfunction are critical underlying pathogenic mechanisms [59]. A strong inverse relationship has been observed between the intake of specific nutrients, such as carotenoids, vitamin C, and zinc, and the risk of optic neuropathy. Higher dietary antioxidant capacity is associated with a lower risk of glaucoma, possibly due to the combined neuroprotective effects of these micronutrients on retinal ganglion cells and their axons [48]. However, evidence drawn from such dietary intake studies is frequently constrained by a lack of standardized protocols for nutritional assessment and significant heterogeneity of study populations, which can lead to inconsistent findings across different observational cohorts. Research suggests that targeted supplementation can augment MPOD levels by restoring macular pigment density, enhancing visual performance and functional outcomes in patients with compromised retinal integrity. Current evidence supports carotenoids as a promising adjunctive nutraceutical approach to be used alongside IOP-lowering therapies rather than as a standalone treatment [60]. The rationale is strong: the glaucomatous retina creates a hostile environment driven by sustained oxidative injury, which depletes endogenous macular carotenoids. Yet, while preclinical data support their neuroprotective benefits, clinical trial results remain controversial and insufficient. Validating this adjunctive therapy is hindered by a limited number of randomized controlled trials, typically constrained by small sample sizes, short follow-up durations, inconsistent supplementation doses, and variable outcome measures. Therefore, rigorously controlled prospective studies are essential to confirm whether this approach provides definitive clinical benefits. Skin carotenoid levels may be a reliable non-invasive biomarker for glaucoma management. Studies have demonstrated an inverse correlation between skin carotenoid levels and AGEs, which is pronounced in patients with exfoliation glaucoma (EG), who typically exhibit higher systemic oxidative stress markers compared to patients with primary open-angle glaucoma [61]. In a cross-sectional study, patients with normal-tension glaucoma (NTG) had lower serum retinol concentrations. Serum retinol was positively correlated with optic nerve sheath diameter in glaucoma patients and was not associated with other demographic or ophthalmic parameters in the NTG patients. Multivariate logistic regression showed that serum retinol was associated with NTG incidence, suggesting that serum retinol may be a new option for the diagnosis and monitoring of the disease [62]. The mechanistic rationale linking specific antioxidants directly to ganglion cell survival still relies heavily on a predominance of preclinical evidence. Further longitudinal clinical trials are necessary to confirm whether these cross-sectional observations can be translated into reliable diagnostic and therapeutic tools.

#### 5.2.2. Optic Neuropathies

Optic neuropathy is a condition leading to vision loss. Clinically, these neuropathies typically present with subacute, bilateral, symmetrical and painless visual impairment. Early visual disturbances include: dyschromatopsia and loss of contrast sensitivity. Moreover, testing often reveals central or centrocecal scotomas. During an ophthalmic examination, the appearance of the optic disc evolves as neuropathy progresses. Observation often reveals disc swelling, loss of the papillomacular bundle, mild hyperaemia, hemorrhage, and later atrophy. These clinical characteristics are similar across inherited optic neuropathies, toxic neuropathies, and malnutrition-related neuropathies. Malnutrition-related neuropathies have been traditionally associated with severe malnutrition in developing regions or chronic alcoholism. The Cuban epidemic of nutritional optic neuropathy highlighted that the depletion of antioxidant carotenoids serves as a critical co-factor in axonal vulnerability, stripping the papillomacular bundle of its protective chemical shield and exacerbating the neurotoxic effects of B-complex vitamin deficiencies. However, a new high-risk group has been identified: patients who have undergone bariatric surgery in the past. Post-surgical malabsorption can lead to deficiencies in micronutrients essential for optic nerve health [63]. Another emerging risk group includes individuals with autism spectrum disorder. They often present with stereotyped behaviors and restricted diets, which often result in malnutrition, for example, as hypovitaminosis A, which is manifested by optic neuropathy [63,64,65]. Clinical cases of autism spectrum disorder-related malnutrition demonstrate that high-dose Vitamin A and Zinc supplementation can rapidly restore retinal function and resolve corneal defects, but they often fail to reverse structural axonal loss, as evidenced by persistent Optical Coherence Tomography (OCT) thinning and impaired Visual Evoked Potentials findings [64,65,66]. This highlights a critical therapeutic gap where the bioactive, antioxidant properties of carotenoids are essential to provide the structural neuroprotection that Vitamin A alone cannot offer. The bioactive properties of carotenoids are equally critical in the context of Toxic Optic Neuropathy. Ethambutol and Isoniazid are first-line agents in tuberculosis treatment. They are known for inducing toxic optic neuropathy. A recent experimental study demonstrated that lutein can effectively mitigate this neurotoxicity and prevent toxic optic neuropathy [66,67]. Ultimately, eyes supplemented with lutein perform significantly better in clinical settings due to their specialized neuroprotective properties and the preservation of synaptic activity, ensuring that the structural integrity of the nerve remains intact even under metabolic stress [60,68].

#### 5.2.3. Neurodegenerative Diseases with Ocular Manifestations

The retina, as an embryological extension of the central nervous system, serves as a non-invasive “window” into the pathological changes occurring in the brain. In many neurodegenerative diseases, ocular manifestations not only mirror cerebral atrophy but also frequently precede clinical systemic symptoms, providing an opportunity for early diagnosis and monitoring through technologies like OCT [69].

Alzheimer’s disease (AD) is characterized by a significant reduction in Retinal Nerve Fiber Layer thickness and a loss of retinal ganglion cells. It closely mirrors the atrophy occurring in the brain tissue. At a molecular level, the retina mirrors pathology through the accumulation of B-amyloid plaques and tau aggregates, which compromise the blood–retinal barrier and trigger neuroinflammation. These structural changes manifest as impaired contrast sensitivity, color vision disturbances, and higher-order visuospatial impairments [70]. The therapeutic potential of carotenoids in AD is supported by clinical evidence. A 12-month randomized clinical trial demonstrated that daily supplementation with 10 mg lutein, 10 mg meso-zeaxanthin, 2 mg zeaxanthin, omega-3 fatty acids, and vitamin E significantly slows the progression of Alzheimer’s disease. This intervention resulted in statistically significant improvements in objective measures of AD severity, in memory and mood [71]. However, interpreting such multi-nutrient interventions is challenging, as the specific efficacy of carotenoids cannot be easily isolated. Furthermore, the long-term Rush Memory and Aging Project revealed through post-mortem brain autopsies of over 500 participants that those with higher dietary intake of lutein, zeaxanthin, and lycopene had significantly less global AD pathology, including a lower density of toxic plaques and tangles [72].

Ocular manifestations of Parkinson’s disease (PD) are characterized by retinal thinning, measurable via OCT. Classic PD typically involves localized peripapillary retinal nerve fiber layer loss, while Atypical PD Syndromes, such as Multiple System Atrophy and Progressive Supranuclear Palsy, exhibit significantly more aggressive and widespread macular thinning. These structural alterations reflect the underlying accumulation of toxic a-synuclein or tau proteins [73,74]. Long-term prevention is also linked to carotenoid status; a prospective study of over 80,000 participants followed for 15 years found that higher intake of B-carotene was associated with a significantly lower risk of developing PD in both men and women [75]. Despite this solid sample size, observational data are inherently limited by a lack of standardized protocols for dietary assessment and the heterogeneity of study populations, which can lead to inconsistent findings across different cohorts.

In Multiple Sclerosis (MS), optic neuritis is a hallmark inflammatory neuropathy affecting 40–60% of patients. Frequently, it is the first clinical sign of systemic autoimmune disease and leads to permanent visual deficits through progressive axonal degeneration in the optic nerve fiber layer [76]. Retinal Nerve Fiber Layer thinning assessed by OCT is a useful marker for assessing MS progression [77]. A clinical trial on patients with Relapsing-Remitting MS showed that daily supplementation with 20 mg of lutein significantly increased carotenoid levels in the serum, skin, and retina after a four-month period. Broad cognitive improvements were not observed, but the specific increase in MPOD was directly associated with boosted attention and spatial memory performance. These findings suggest that the successful accumulation of lutein in the retina may be a functional marker for neuroprotective and cognitive benefits in the MS population [78]. Evidence in MS relies on a very limited number of randomized controlled trials, making it difficult to establish definitive treatment guidelines.

Emerging evidence suggests that AD, Parkinson’s, and glaucoma should be viewed as interconnected age-related neurodegenerative diseases. AD and AMD share some signaling defects, including the presence of B-amyloid in both brain plaques and retinal drusen [79]. This suggests that neuroprotective strategies for the eye may also significantly benefit the brain. Carotenoids, especially lutein, support brain health by acting on several pathways of cellular dysfunction involved in cell damage. Lutein helps reduce oxidative stress, protects mitochondria, and prevents cell death (including ferroptosis). It also limits excitotoxicity, reduces inflammation, and may improve cognitive function in AD patients [80]. Although these mechanistic pathways are biologically plausible, they are primarily supported by a predominance of preclinical evidence, highlighting the gap between theoretical neuroprotection and proven clinical efficacy. These benefits are further supported by neuroimaging studies using functional Magnetic Resonance Imaging (fMRI). Higher levels of these carotenoids are associated with increased neural efficiency in fMRI, preventing the compensatory over-activation in the occipital and frontal cortexes often seen in age-related decline [81]. The evidence confirms that neurodegenerative diseases manifest in the eye, where the retina reflects the brain’s pathological state. As carotenoids demonstrate potent neuroprotective effects against these disorders, they play a dual role in protecting both cognitive function and ocular integrity. Carotenoids act as a neuroprotective shield targeting the eye-brain axis and represent a promising strategy to mitigate the visual and neurological decline associated with these conditions.

### 5.3. Lens Disorders

Cataracts are one of the leading causes of blindness and visual impairment worldwide in both developed and developing countries, and significantly reduce patients’ quality of life [82,83]. Depending on its anatomical structure, cataracts are classified as cortical cataracts, where the outer layer of the lens is clouded, nuclear cataracts, where the clouding affects the inner core, and posterior subcapsular cataracts, where the clouding affects the superficial area beneath the capsule at the back of the eye [17]. Over 100 million people worldwide are affected by cataracts, with nearly 70 million cases resulting in bilateral blindness or moderate and severe visual impairment. The prevalence of cataracts increases with age. Main risk factors for the development of cataracts include ageing, smoking, exposure to Ultraviolet (UV) light and diabetes [82,83]. Furthermore, other risk factors for cataracts include gender (with a higher risk among women), a lower level of education, high blood pressure and alcohol consumption [83,84]. The lens is constantly exposed to oxidative stress from internal sources such as metabolic activity and inflammation, as well as external sources such as UV, ionising and gamma radiation, which is the main cause of lens opacification.

ROS causes oxidation, deamination, tryptophan derivatisation and cross-linking of crystalline proteins. UV-induced ROS oxidizes the amino acids in alpha-crystallin, leading to cross-linking and oligomerisation of the proteins. This results in the formation of light-scattering protein aggregates. In cataractous lenses, increased levels of lipid hydroperoxides have been reported, arising from ROS-mediated peroxidation of polyunsaturated fatty acids (PUFAs), ultimately leading to impairment of cellular membrane integrity. Furthermore, ROS induces apoptosis in lens epithelial cells (LECs), which is one of the mechanisms underlying cataract development. Lens cells are protected from this process by epigallocatechin gallate (EGCG), mini-chaperones and N-acetylcarnosine [85,86]. The numerous mitochondria present in the epithelial cells of the lens and in the young lens fibres account for approximately 90% of oxygen consumption in the lens. Redox homeostasis is disrupted with age, resulting in the accumulation of damage to lens cell biomolecules, including proteins, lipids and DNA, which leads to lens cell dysfunction and the development of pathologies, including lens opacity [87]. Carotenoids are antioxidant compounds whose presence in the lens contributes to protection against oxidative damage. The lens consists of lutein and zeaxanthin, which are presumably delivered from the body pool to the epithelial layer of the lens. Lutein and zeaxanthin protect the eye’s structures from oxidative stress and mitochondrial damage, neutralise free radicals and ROS, and absorb high-energy, short-wavelength light, thereby protecting the lens from photochemical damage and development of cataracts [17,24,88,89]. Studies indicate that patients with cataracts have elevated levels of pro-oxidants in their serum and reduced levels of antioxidants [17]. The results of the meta-analysis of observational studies showed that a reduction in the incidence of nuclear cataracts was significantly associated with high serum concentrations of lutein and zeaxanthin, particularly in the case of zeaxanthin. With regard to cortical and subcapsular cataracts, no significant associations were found between these types of cataract and blood levels of zeaxanthin or lutein. However, given the numerous limitations of the studies and meta-analyses conducted, long-term, large-scale, prospective, controlled intervention trials are needed to further clarify the effect of lutein and zeaxanthin on the development of age-related cataracts [88]. Epidemiological studies have identified a link between higher dietary intake of carotenoids and a reduced risk of cataracts. They suggest that diets rich in lutein and zeaxanthin—whether through food intake or the use of supplements—may be crucial in preventing or delaying the development of cataracts. However, in order to conclusively establish the validity of carotenoid supplementation, research into their metabolism is required [90].

### 5.4. Ocular Surface and Anterior Segment Diseases

#### 5.4.1. Dry Eye Disease

Dry eye disease (DED) is a multifactorial ocular surface disorder characterized by a loss of tear film homeostasis and the presence of clinical symptoms such as discomfort, visual disturbances, burning, and a sensation of dryness. The main pathogenic mechanisms include tear film instability and hyperosmolarity, inflammatory changes, and damage to the ocular surface epithelium—creating a vicious cycle in which tear film abnormalities exacerbate inflammatory processes, which in turn worsen ocular surface dysfunction, leading to further disease progression [91,92]. Recent studies emphasize that ocular surface inflammation is a key component of the pathophysiology of DED. This process involves the activation of inflammatory signals, the release of pro-inflammatory cytokines, and the recruitment of immune cells, leading to epithelial damage and further tear film dysfunction. In DED models, enhanced signaling pathways associated with MAPK and NF-κB are observed, which induce the expression of mediators such as IL-1β, IL-6, and TNF-α, which are key in driving the inflammatory response and exacerbating damage to ocular surface tissues [92,93]. Furthermore, metabolic changes in the epithelial cells of the ocular surface are closely linked to inflammation in DED. Studies have shown that, as the disease progresses, there is a remodeling of glucose, lipid, and amino acid metabolism in epithelial cells, which is associated with the metabolic activation of immune cells and the production of pro-inflammatory mediators. This metabolic reprogramming provides the energy and metabolites necessary for cytokine synthesis, further exacerbating local inflammation [92]. In the context of inflammation, immuno-inflammatory processes involve the activation of cytokine-related signaling pathways, lymphocyte recruitment, and the overproduction of pro-inflammatory mediators, leading to further damage and apoptosis of ocular surface epithelial cells. These mechanisms underscore the importance of the immune response and chronic inflammation in the etiology of DED [94,95,96]. Furthermore, DED is associated with metabolic and immunological changes on the ocular surface, which include disturbances in lipid, glucose, and amino acid metabolism, as well as modifications in cellular signaling pathways. These changes co-occur with inflammatory changes and indicate that DED is not only a disorder of the tear film but also a complex dysfunction of tissues and the immune response [92,96].

#### 5.4.2. Meibomian Gland Dysfunction

Meibomian gland dysfunction (MGD) is a chronic disorder of the eyelid sebaceous glands and is the primary cause of evaporative dry eye (evaporative DED). The condition is characterized by a reduced quantity and altered quality of meibum secretion, which leads to destabilization of the lipid layer of the tear film, increased tear evaporation, hyperosmolarity, and secondary inflammation of the ocular surface. These changes result in cyclic damage to the ocular surface epithelium and exacerbation of DED symptoms, such as burning, dryness, discomfort, and visual disturbances [97,98]. The prevalence of MGD is high in clinical and general populations; however, its diagnosis depends on the diagnostic criteria used. Multicenter analyses have shown that the prevalence of MGD in DED varies depending on the clinical definition, and the degree of gland atrophy increases with age and may vary by gender [99]. Pathophysiologically, MGD results from molecular disturbances in the homeostasis of the Meibomian glands, leading to metabolic failure of meibocyte cells, changes in the composition of meibum lipids, and chronic inflammation. As a result, destabilization of the tear film, increased evaporation, and secondary inflammation of the ocular surface are observed, which exacerbates the clinical symptoms of the disease [100,101]. Taken together, these processes underscore that MGD is a complex, multifactorial disorder involving anatomical, metabolic, and immuno-inflammatory changes, which consequently lead to symptoms of DED [97,99].

#### 5.4.3. Allergic and Inflammatory Ocular Surface Disorders

Allergic and immuno-inflammatory diseases of the ocular surface constitute a significant proportion of clinical entities in ophthalmology, including allergic conjunctivitis (AC)—both seasonal and perennial forms—atopic keratoconjunctivitis, and other immuno-inflammatory conditions. They serve as a practical model of immune response disorders on the ocular surface, in which Type I hypersensitivity reactions involving immunoglobulin E (IgE), mast cells, Type 2 T lymphocytes, and numerous pro-inflammatory cytokines play a key role [102,103]. In allergic conjunctivitis, sensitization to allergens leads to the binding of IgE to FcεRI receptors on mast cells, which, upon subsequent contact with the allergen, triggers mast cell degranulation and the release of inflammatory mediators such as histamine, leukotrienes, and cytokines such as interleukin 4 (IL-4), interleukin 5 (IL-5), interleukin 13 (IL-13), which initiate and sustain the inflammatory response on the ocular surface, leading to initial symptoms such as itching, redness, tearing, and swelling [103,104]. The immunological pathogenesis of ocular surface allergies is multifaceted. It involves both IgE-mediated reactions and chronic inflammatory responses involving Th2 cells and other effector cell populations, which produce characteristic cytokines and chemokines that enhance the infiltration of inflammatory cells into the conjunctiva and epithelium [105]. The initial phase of the immune response to allergens includes the classic immediate phase mediated by mediators released from mast cells, followed by a late phase in which there is further recruitment of eosinophils, T lymphocytes, and other inflammatory cells, as well as the continued secretion of cytokines and chemokines, which sustain chronic inflammation and structural changes in the conjunctiva [102,105]. Allergic inflammatory reactions on the ocular surface also have clinical and social significance: these disorders are common in the general population, with a significant impact on the patient’s quality of life, and their symptoms can cause subjective discomfort, reduced productivity, and a predisposition to complications, particularly when combined with other ocular surface conditions, such as dry eye disease or Meibomian gland dysfunction [103]. In addition, the treatment of allergic conjunctivitis requires a comprehensive approach—ranging from the diagnosis of immunological and allergic mechanisms, through the stabilization of the tear film mucosa, to the use of topical antihistamines and anti-inflammatory therapies, as well as, increasingly, targeted immunomodulatory therapies that block specific cytokines and their receptors to reduce chronic inflammation of the ocular surface [103,104,106]. To facilitate comparison across different ocular conditions, the principal clinical studies evaluating carotenoids in ophthalmology are summarized in Table 2.

### 5.5. Developmental Eye Disorders

Recent studies on prenatal ocular development have revealed that the accumulation of carotenoids within the human vitreous body is distinct and transient. In contrast to the adult vitreous, which is transparent and carotenoid-free, the fetal vitreous undergoes biochemical transformation during the second trimester. Around weeks 14–16 of gestation, the vitreous changes from a transparent gel to a subtle yellow hue. This is followed by a peak carotenoid accumulation period between 16 and 23 weeks, marked by high concentrations of lutein. Subsequently, between 24 and 28 weeks, carotenoid levels drop sharply as the vitreous becomes colorless, and by the 30th week of gestation, carotenoids are no longer detectable. Analysis using high-performance liquid chromatography and tandem mass spectrometry, researchers confirm that lutein is the predominant carotenoid in the developing fetal eye, while zeaxanthin is absent. This contrasts with the adult macula, where both lutein and zeaxanthin are essential components. The distribution of lutein and its metabolites varies across ocular structures. The vitreous, retina, and RPE contain both native lutein and its oxidized forms; the lens and ciliary body-iris complex contain only the oxidized metabolites. The presence of lutein during the second trimester suggests a temporally regulated role in eye development. Its disappearance coincides with the regression of the hyaloid vascular system and maturation of the vitreous body into its transparent, adult-like form [106,108,109,110]. Beyond the structural functions of xanthophylls, early eye formation is also governed by all-trans retinoic acid (RA). Acting through RAR signaling, RA orchestrates communication between the optic vesicle and the prospective lens ectoderm. This process begins with an initial activation phase, where RA induces the transcription factors Lhx2, Mab21l2, Rx, and Hes1. These factors then drive the upregulation of morphogens, including BMP4 and BMP7. Finally, during terminal activation, the activation of SIX3 and PAX6 leads to the definitive formation of the lens placode [111]. After birth, carotenoid supply is primarily maintained through breastfeeding, which is a more efficient source of these compounds than infant formula. While carotenoids in breast milk decline over time, lutein remains relatively stable, accounting for a substantial proportion of total carotenoid content in mature milk. This sustained supply is important because newborns possess limited endogenous antioxidant capacity. The selective retention of lutein in human milk, despite a marked reduction in other carotenoids such as lycopene, suggests an evolutionarily conserved mechanism ensuring continuous delivery of this critical protective factor to the developing retina during early life [109,111].

### 5.6. Pediatric Eye Disorders

The increasing use of digital devices among children has led to a rise in Computer Vision Syndrome (CVS), making it an emerging pediatric health concern. A randomized, double-blind, placebo-controlled trial in children aged 10–14 years demonstrated that daily supplementation with 4 mg of astaxanthin for 84 days significantly improved CVS symptoms. Participants showed a 20% greater reduction in CVS questionnaire scores and a 27% decrease in visual fatigue compared to the placebo group. Additionally, improvements in stereopsis and pupillary light reflex suggest enhanced visual processing and neuromuscular recovery. Increased tear production further indicates benefits in alleviating screen-related dry eye. Importantly, astaxanthin was well tolerated, with no adverse effects on visual acuity or systemic health [107]. In contrast, the role of other carotenoids, such as lutein and zeaxanthin, appears to be more complex and condition-dependent. Current evidence indicates no significant association between lutein and zeaxanthin intake and the risk, prevalence, or severity of refractive errors, including myopia and astigmatism [112]. Similarly, prospective data suggest that maternal and early childhood intake of lutein and zeaxanthin does not directly correlate with visual acuity or contrast sensitivity in healthy children, although some subgroup-specific effects have been observed depending on maternal factors [113]. Despite their well-established antioxidant properties, lutein and zeaxanthin supplementation have not demonstrated efficacy in the primary prevention of retinopathy of prematurity (ROP). A systematic review of randomized controlled trials in preterm infants found no significant reduction in ROP incidence, though supplementation was safe in this population [114]. Emerging evidence suggests that systemic carotenoid status during pregnancy may influence early visual development. The GUSTO cohort study found that higher maternal plasma levels of lutein and zeaxanthin were associated with a lower risk of poor visual acuity in children at three years of age, highlighting the importance of objective maternal antioxidant status for early visual outcomes [115]. Recent interventional studies indicate that lutein supplementation may have structural rather than functional effects. A randomized controlled trial showed that lutein esters attenuated choroidal thinning in children. However, no significant effects were observed on axial length or refractive error progression within the study period, indicating that longer-term studies are needed to confirm clinical relevance [116]. An observational study suggests that MPOD may not be a direct determinant of visual performance in healthy pediatric populations [116]. Studies have shown no significant associations between MPOD and visual acuity, contrast sensitivity, or refractive status, potentially due to relatively homogeneous dietary patterns and adequate baseline nutrition in the studied cohorts. These cohorts consisted of children with emmetropia or mild refractive errors and moderate-to-high adherence to a Mediterranean diet, lacking a nutritionally deficient group for comparison. Furthermore, while correlations between MPOD and parameters like contrast sensitivity are well-documented in adults, such functional effects are too subtle to detect in children [117]. Their already optimal baseline visual function creates a “ceiling effect”, where higher macular pigment levels do not demonstrably enhance basic visual metrics [81,117]. Therefore, early macular carotenoid accumulation primarily serves a prophylactic role. It establishes a protective reservoir that provides cumulative protection against oxidative damage over a lifespan [44,81]. Clinical evidence indicates that maternal carotenoid intake during pregnancy and lactation directly influences infant serum levels. Higher MPOD values in children have been associated with improved cognitive performance [117]. However, given that dietary intake of carotenoid-rich foods is often insufficient among both women and children, targeted nutritional strategies, including supplementation and dietary modification, may be necessary to support optimal visual and neurodevelopmental outcomes.

## 6. Conclusions

Carotenoids play a vital role in protecting the eyes, exhibiting multifaceted biological effects, including antioxidant, anti-inflammatory and neuroprotective properties. Their specific role stems from their selective accumulation in the structures of the eye, particularly in the macula, where they help both prevent the development of oxidative stress and neutralise its effects. The ability of carotenoids to absorb blue light and directly scavenge reactive oxygen species translates into a reduction in cellular damage in the retina, which is a key element in the pathogenesis of many ophthalmic diseases. Furthermore, their influence on the modulation of inflammatory pathways, mitochondrial function and the stability of the blood–retinal barrier underscores their importance in maintaining ocular tissue homeostasis. Another important aspect is the bioavailability of carotenoids, which depends on the presence of fats in the diet, the dietary source, and intestinal transport mechanisms involving specific proteins such as SR-BI and NPC1L1. These factors determine their serum concentration and the efficiency of distribution to ocular tissues, which directly influences their biological activity. Available clinical and epidemiological data suggest that an adequate intake of carotenoids, particularly lutein and zeaxanthin, may help reduce the risk of developing and slow the progression of retinal diseases, including age-related macular degeneration. However, their effects depend on many factors, such as interactions with other dietary components, genetic factors and the stage of the disease. Despite the increasing number of studies confirming the beneficial effects of carotenoids on eye health, there remains a need for high-quality clinical trials to definitively establish their therapeutic efficacy, optimal doses and long-term safety.

In summary, carotenoids play a key role in protecting the structures of the eye and may constitute an important element of preventive strategies and adjunctive treatments for eye diseases. However, their full clinical potential requires further systematisation and confirmation in future studies.

## Figures and Tables

**Table 1:** Summary of the major carotenoids, their molecular targets, mechanisms of action, and associated ocular diseases [33,35,37].

Carotenoid	Major Molecular Targets	Proposed Mechanisms of Action	Associated Ocular Diseases
Lutein	Nrf2, NF-κB, MAPK (ERK/JNK/p38), VEGR, BDNF, Occludin	Blue light filtration, scavenging of reactive oxygen species (ROS); activation of antioxidant defense; inhibition of NF-κB and MAPK signaling; reduction in oxidative stress and inflammation; stabilization of the blood–retinal barrier; neuroprotection; inhibition of angiogenesis	Age-related macular degeneration (AMD); diabetic retinopathy (DR); glaucoma; retinal ischemia/reperfusion injury; uveitis; cataract
Zeaxanthin	Nrf2, NF-κB, ROS, mitochondrial pathways	Blue light filtration; antioxidant activity; inhibition of lipid peroxidation; reduction in oxidative stress; preservation of mitochondrial function; anti-inflammatory effects	Age-related macular degeneration (AMD); diabetic retinopathy (DR); cataract
Meso-zeaxanthin	Macular pigment; ROS	Enhancement of macular pigment optical density (MPOD); blue light filtration; antioxidant protection of photoreceptors; improvement of visual performance	Age-related macular degeneration (AMD); age-related visual impairment

**Table 2:** Major clinical trials evaluating carotenoid supplementation in ocular diseases [42,44,45,52,59,70,77,87].

Disease	Clinical Study	Supplementation	Study Population	Major Outcomes	References
Age-related macular degeneration (AMD)	AREDS2	Lutein 10 mg + zeaxanthin 2 mg/day	Intermediate AMD	Reduced progression to advanced AMD; β-carotene replacement maintained efficacy while reducing lung cancer risk in smokers.	[45]
Geographic atrophy	AREDS2 secondary analysis	Lutein 10 mg + zeaxanthin 2 mg/day	Non-central GA	Slower lesion expansion toward the fovea.	[45]
AMD	AREDS/AREDS nutritional analyses	High dietary lutein, zeaxanthin and β-carotene	>7700 participants	Lower risk of late AMD and neovascular complications.	[42,44]
Diabetic retinopathy	MPOD supplementation study	Lutein 10–20 mg/day	Patients with diabetes	Faster increase in MPOD with 20 mg/day.	[52]
Alzheimer’s disease	Randomized clinical trial	Lutein 10 mg + meso-zeaxanthin 10 mg + zeaxanthin 2 mg + ω-3 + vitamin E	Alzheimer’s disease	Improved cognition, mood and disease severity.	[70]
Multiple sclerosis	Clinical trial	Lutein 20 mg/day	RRMS patients	Increased serum, skin and retinal carotenoid levels; higher MPOD associated with better attention and spatial memory.	[77]
Glaucoma	Clinical studies	Lutein/zeaxanthin	Glaucoma patients	Increased macular pigment optical density and improved visual function; supports adjunctive neuroprotection.	[59]
Cataract	Meta-analysis	Higher serum lutein/zeaxanthin	Observational cohorts	Higher circulating lutein/zeaxanthin associated with reduced risk of nuclear cataract.	[87]
Computer Vision Syndrome (CVS)	Randomized, double-blind, placebo-controlled trial	Astaxanthin	Children aged 10–14 years	Reduced visual fatigue and improved CVS symptoms.	[107]

## Data Availability

No new data were created or analyzed in this study. Data sharing is not applicable to this article.

## Abbreviations

AC	allergic conjunctivitis
AD	Alzheimer’s disease
AGEs	advanced glycation end products
AMD	Age-related macular degeneration
ARE	Antioxidant Response Element
AREDS and AREDS2	Age-Related Eye Disease Studies
BDNF	Brain-derived neurotrophic factor
BRB	Blood–Retinal Barrier
CRP	C-reactive protein
CVS	Computer Vision Syndrome
DED	Dry Eye Disease
DM	Diabetes mellitus
DR	Diabetic Retinopathy
DNA	Deoxyribonucleic acid
EG	Exfoliation glaucoma
EGCG	Epigallocatechin gallate
EMT	Epithelial–mesenchymal transition
ERK	Extracellular Signal-Regulated Kinases
fMRI	Functional Magnetic Resonance Imaging
GA	Geographic Atrophy
GSTP1	Glutathione S-transferase Pi 1
HDL	High-Density Lipoprotein
HO-1	Heme Oxygenase-1
ICAM-1	Intercellular Adhesion Molecule-1
IgE	Immunoglobulin E
iNOS	Inducible Nitric Oxide Synthase
IL-1β	Interleukin 1β
IL-5	Interleukin 5
IL-6	Interleukin 6
IL-8	Interleukin 8
IL-13	Interleukin 13
IOP	Intraocular pressure
JNK	c-Jun N-terminal Kinases
KEAP1	Kelch-like ECH-associated protein A
LDL	Low-Density Lipoprotein
LECs	Lens epithelial cells
MAPK	Mitogen-Activated Protein Kinases
MCP-1	Monocyte Chemoattractant Protein 1
MGD	Meibomian gland dysfunction
MPOD	Macular pigment optical density
MS	Multiple Sclerosis
NADPH	Reduced form of Nicotinamide Adenine Dinucleotide Phosphate
NF-*κ*B	Nuclear Factor kappa B
NQO-1	NADPH Quinone Oxidoreductase 1
NRF2	Nuclear factor erythroid 2-related factor 2
NPC1L1	Niemann-Pick C1-Like 1
NTG	Normal-tension glaucoma
OCT	Optical Coherence Tomography
PUFAs	Polyunsaturated fatty acids
RA	Retinoic acid
RPE	Retinal pigment epithelium
RP	Retinitis pigmentosa
ROS	Reactive oxygen species
ROP	Retinopathy of prematurity
SR-BI	Scavenger receptor class B type I
STGD1	Stargardt disease type 1
StARD3	StAR-related lipid transfer domain protein 3
TNF-α	Tumor Necrosis Factor alpha
UV	Ultraviolet
VEGF	Vascular Endothelial Growth Factor
